# Retraction Note: Plant disease recognition using residual convolutional enlightened Swin transformer networks

**DOI:** 10.1038/s41598-026-42011-2

**Published:** 2026-03-09

**Authors:** Ponugoti Kalpana, R. Anandan, Abdelazim G. Hussien, Hazem Migdady, Laith Abualigah

**Affiliations:** 1 Department of Computer Science Engineering, Vels Institute of Science Technology and Advanced Studies, Chennai, Tamil Nadu 600117 India; 2 Department of Computer and Information Science, Link\xF6ping University, Link\xF6ping, Sweden; 3 CSMIS Department, Oman College of Management and Technology, 320 Barka, Oman; 4 Artificial Intelligence and Sensing Technologies (AIST) Research Center, University of Tabuk, 71491 Tabuk, Saudi Arabia; 5 Computer Science Department, Al Al-Bayt University, Mafraq, 25113 Jordan; 6 Hourani Center for Applied Scientific Research, Al-Ahliyya Amman University, Amman, 19328 Jordan; 7 MEU Research Unit, Middle East University, Amman, 11831 Jordan; 8 Department of Electrical and Computer Engineering, Lebanese American University, Byblos, 13-5053 Lebanon; 9 School of Computer Sciences, Universiti Sains Malaysia, 11800 George Town, Penang Malaysia; 10 School of Engineering and Technology, Sunway University Malaysia, 27500 Petaling Jaya, Malaysia; 11 Applied Science Research Center, Applied Science Private University, Amman, 11931 Jordan; 12 College of Engineering, Yuan Ze University, Taoyuan, Taiwan; 13 Faculty of Science, Fayoum University, Fayoum, Egypt

Retraction of: *Scientific Reports* 10.1038/s41598-024-56393-8, published online 15 April 2024

The Editors have retracted this Article.

After publication, concerns were raised about the non-standard terminology used in the Article. Further investigation by the Editors uncovered authorship irregularities. The Authors were contacted for explanations but their reply did not resolve the concerns. The Editors have therefore lost confidence in the contents of this Article.

All Authors disagree with the retraction.

